# Differences in surgical site infection rates by state according to state-mandated operating room air changes per hour

**DOI:** 10.1186/s13756-025-01631-5

**Published:** 2025-10-08

**Authors:** Andrew Atkinson, Jonas Marschall, Jason P. Burnham

**Affiliations:** 1 Division of Infectious Diseases, Washington University in St. Louis School of Medicine, 4523 Clayton Avenue, Campus Box 8051, St. Louis, MO 63110 USA; 2 Division of Infectious Diseases, University of Arizona College of Medicine, Phoenix, Phoenix, AZ USA

**Keywords:** Air changes per hour, Operating rooms, Surgical site infections, State mandates

## Abstract

**Background:**

Air changes per hour (ACH) in operating rooms (ORs) are energy intensive, and optimal air change settings are not known.

**Objectives:**

We sought to explore whether there is a relationship between surgical site infections (SSIs) across states based on their state-mandated ACHs.

**Design:**

Ecological, descriptive, cross-sectional study of publicly reported SSI data in the United States.

**Methods:**

Wilcoxon test was used to investigate differences between SSI rates for specific surgery types between ACH mandate levels (15 and 20 ACH). Uni- and multivariable Poisson models at the state level were fitted to estimate differences in SSI rates for each surgery type.

**Results:**

OR ACH mandates and SSIs were positively correlated for C-sections and spinal fusion; negatively correlated for colon and laminectomy surgery.

**Conclusion:**

For most surgery types, there is no correlation between state-mandated OR ACH. Further studies are needed to determine what changes to mandates can be made safely and effectively.

**Supplementary Information:**

The online version contains supplementary material available at 10.1186/s13756-025-01631-5.

## Introduction

Climate change stemming from greenhouse gas emissions is one of the most important public health issues of the twenty-first century [1]. Healthcare itself is responsible for 8.5% of U.S. greenhouse gas emissions and similar fractions of toxic air pollution (from fossil fuel combustion) [2]. Of US healthcare’s carbon footprint, ~ 40% is from heating, ventilation, and air conditioning (HVAC) systems. As 70–97% of energy use in operating rooms (ORs) is related to HVAC systems [3, 4], reducing HVAC use could be one way to reduce healthcare’s carbon footprint.

There are significant evidentiary gaps regarding optimal air change settings for healthcare systems, including in ORs [5]. A recent review concluded further investigation was required on optimal air changes per hour (ACH) in ORs, as evidence to support 20 ACH was mixed and pulled from few studies [5]. Air changes refers to complete replacement of air in a space. Within hospitals, ORs have the highest ACH requirement, and therefore, have the largest potential for energy savings. Although risk factors for SSIs are myriad (including patient factors such as age, sex, and medical comorbidities including diabetes and blood glucose control, surgical factors such as contamination and duration of surgery), we sought to evaluate one component of the SSI risk profile, ACH in ORs, to explore the relationship between ACH and SSIs. As states within the US have different ACH requirements for ORs, we sought to estimate differences in SSIs across states based on their state-mandated ACHs.

## Methods

This is a descriptive ecological study with cross-sectional data.

### Data source

Facility guidelines institute (FGI) maps for 2018 (based on American Society of Heating, Refrigerating, and Air-Conditioning Engineers [ASHRAE] standards), 2018 state law, or https://up.codes were used to determine ACH mandates for calendar year 2018. Not all states had available information on OR ACH requirements, and for those without data, the states were excluded. Publicly reported SSI data [6] were utilized for 2018 for SSIs, and SSI rates were calculated as the number of observed SSIs over total number of procedures for each surgery type for each state. To attempt to adjust for differences in population risk for SSI, other data from 2018 incorporated into the analytic models included state population [7]. state gross domestic product (GDP) in millions of US dollars [8], and number of persons on Medicaid insurance by state [9]. Surgeries analyzed included the following categories reportable to the National Healthcare Safety Network (NHSN): colon, hysterectomy, hip, knee, rectal, vaginal hysterectomy, coronary artery bypass graft, other cardiac, bypass, aneurysm, caesarean section, spinal fusion, laminectomy, gallbladder, and open fracture. Acute care hospitals are required to report data on abdominal hysterectomies and colon procedures to NHSN; all other procedure reporting is optional at a national level.

### Analysis

Descriptive statistics for each state, including SSI rate and air quality metrics, were summarized as number (percentage) for categorical data and median (inter-quartile range) for continuous data. We used box plots for each surgery type to visualize state SSI rates, stratified by ACH mandates. We included those with missing ACH data to determine if such states had systematically different SSIs rates. The Wilcoxon test was used to investigate differences between SSI rates for specific surgery types between ACH mandate levels (15 and 20). Uni- and multivariable Poisson models at the state level were fitted to estimate differences in SSI rates for each surgery type with numerator of the dependent variable the number of SSIs, the log_10_ number of surgeries as the dominator, and adjusted for ACH mandate (ACH-15 [reference] and ACH-20), state population, GDP and percentage of persons on Medicaid (proxies for socioeconomic status) as independent variables. For each surgery type, univariable predictors statistically significant at the 5% level were included in multivariable models.

Statistical significance level of 5% was used throughout. Analyses were performed in R version 4.2.2 [10]]. The incidence rate ratio (IRR) was the relative measure of association used null.

## Results

In 2018, acute care hospitals reported 2,808,659 procedures to NHSN, and 21,265 infections. ACH mandate data were available for 47 states (Hawaii, Ohio, and Wisconsin had no data)—11 states had a mandated minimum of 15 ACH, 36 had a minimum of 20 ACH (Table S1). In univariate analyses, there were no statistically significant differences between 15 and 20 ACH states except for Caesarean sections (C-section), for which 15 ACH states had lower SSI rates (*p* = 0.04) (Table S1).

**Fig. 1:**
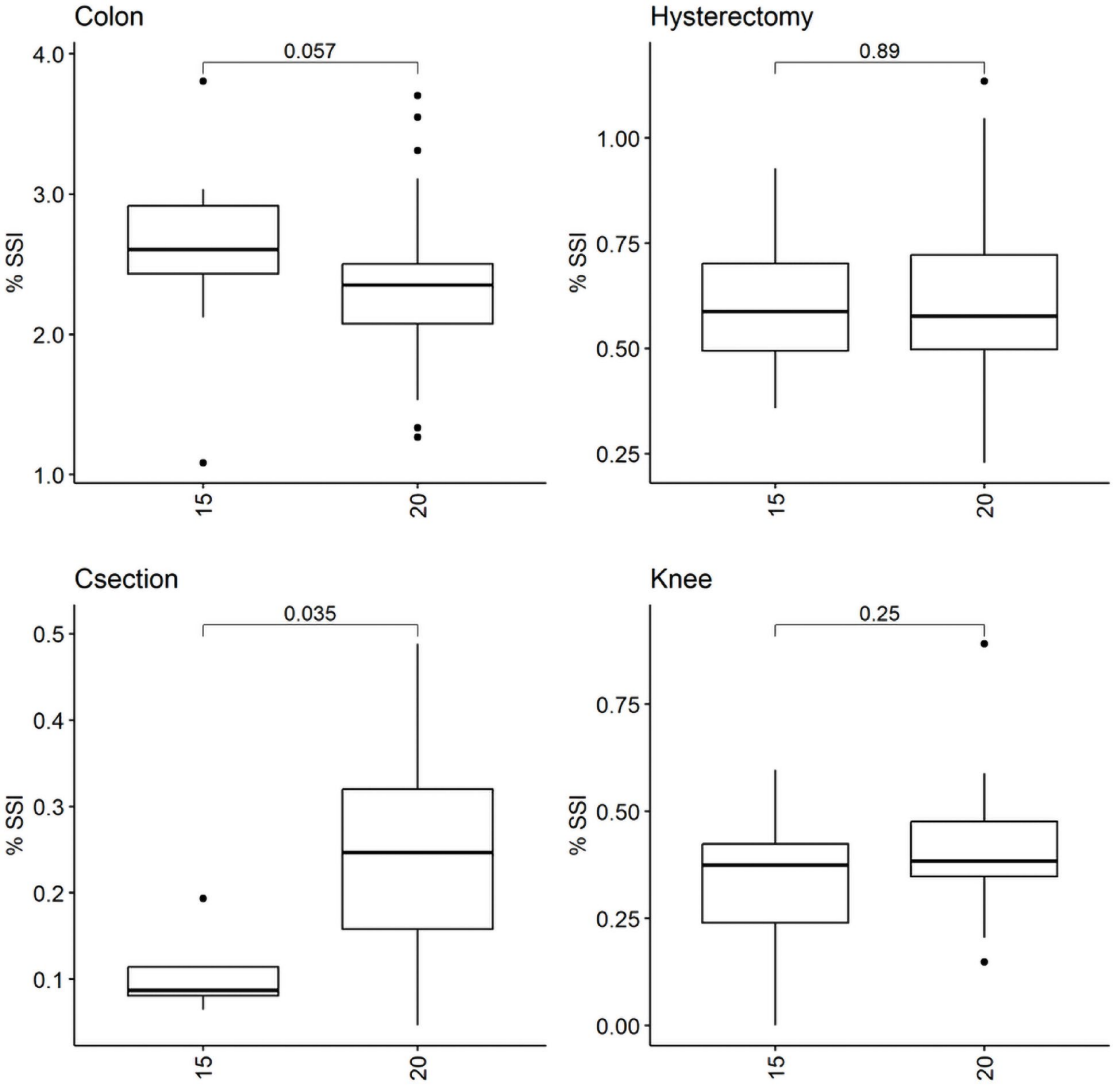
SSI rates for surgery types, stratified by ACH mandates; *p*-values are for the comparison of rates between the main mandate types from the Wilcoxon non-parametric test

For NHSN-defined surgery types in multivariable analysis, higher state OR ACH mandates were associated with increased SSI risk for C-section and spinal fusion, decreased risk for colon surgery (Fig. 1 and Table S2), and no change in risk for other surgeries. In Poisson models explicitly taking into consideration the number of interventions, the C-section trend was confirmed in univariable analyses (incidence rate ratio [IRR] 1.9 for ACH-20 vs. ACH-15, 95% confidence interval [CI], [1.1,3.2], *p* = 0.01), with ACH-20 increasing SSI incidence for spinal fusion in adjusted models (aIRR 1.9 [1.5,2.5], *p* < 0.001), but decreasing SSI incidence for colon (aIRR 0.88 [0.82,0.94], *p* < 0.001) and laminectomies (IRR 0.6, [0.4, 1.0], *p* = 0.03). Box plots of other NHSN surgery types compared by ACH mandate are in Figure S1.

## Discussion

Our study is the first to correlate state-mandated OR ACHs and publicly-reported SSI rates for NHSN-reportable surgeries. We find OR ACH mandates and SSIs are positively correlated for C-sections and spinal fusion (i.e. ACH-20 mandate is associated with more SSIs) and a negative correlation for colon and laminectomy surgery (i.e. ACH-20 mandate associated with fewer SSIs). Eleven NHSN-reportable surgeries had no significant SSI/ACH association.

Our study has several limitations. Our analysis correlates state-level SSI rates and OR ACH mandates. State mandates specify minimum ACH and we cannot determine actual OR ACH. For three states, no OR ACH data were available, which could lead to selection bias. Data needed from future studies include actual OR ACHs used and whether these impact SSIs. If OR ACH are indeed correlated with SSIs, deep dives will be required to generate hypotheses for why ACH have a differential effect on SSIs by surgery type. Further studies with patient and hospital-level data are warranted to explore these correlations. Such studies would also need to consider the following types of data (which we did not have access to for the present analysis): environmental factors associated with ORs (e.g. cleaning/disinfection, movement of persons, etc.) as well as patient-level considerations. The associations we presently find between ACH mandates and SSI rates of various surgery types may be due to unmeasured confounders which need to be studied in a dataset with more granular data on patient and OR-specific information.

## Conclusion

In a first of its kind hypothesis-generating study, we have herein correlated state-mandated OR ACHs and publicly-reported SSI rates for NHSN-reportable surgeries. Large scale studies with multiple hospital systems which incorporate SSI risk factors, multi-component considerations of OR design and airflow, and patient-level SSI data will be required to fully understand the connection between ACH and SSI.

With climate change becoming increasingly severe, carbon emission reductions must be implemented wherever possible. Should the association between ACH (i.e. high energy use states) and SSIs reveal that a lower energy utilization state does no patient harm, then reducing OR ACH requirements may be another step forward in greening healthcare. Further studies are presently required before we know what changes to mandates can be made safely and effectively.

## Supplementary Information

Below is the link to the electronic supplementary material.

Supplementary Material 1

## Data Availability

No datasets were generated or analysed during the current study.

## References

[1] Watts N, Amann M, Ayeb-Karlsson S, Belesova K, Bouley T, Boykoff M, et al. The lancet countdown on health and climate change: from 25 years of inaction to a global transformation for public health. Lancet. 2018;391(10120):581–630. 29096948 10.1016/S0140-6736(17)32464-9

[2] Eckelman MJ, Huang K, Lagasse R, Senay E, Dubrow R, Sherman JD. Health care pollution and public health damage in the United States: an update. Health Aff (Millwood). 2020;39(12):2071–9. 33284703 10.1377/hlthaff.2020.01247

[3] Thiel CL, Eckelman M, Guido R, Huddleston M, Landis AE, Sherman J, et al. Environmental impacts of surgical procedures: life cycle assessment of hysterectomy in the United States. Environ Sci Technol. 2015;49(3):1779–86. 25517602 10.1021/es504719g PMC4319686

[4] MacNeill AJ, Lillywhite R, Brown CJ. The impact of surgery on global climate: a carbon footprinting study of operating theatres in three health systems. Lancet Planet Health. 2017;1(9):e381–8. 29851650 10.1016/S2542-5196(17)30162-6

[5] Mousavi EL, R; Betz, F; Grosskopf, K. Academic Research to Support Facility Guidelines Institute & ANSI/ASHRAE/ASHE Standard 170: ASHRAE CO‐RP‐03. 2019.

[6] Centers for Disease Control and Prevention. 2018 National and State HAI Progress Report SIR Data. 2018.

[7] Bureau of the Census. Annual Estimates of the Resident Population for the United States, Regions, States, and Puerto Rico: April 1, 2010 to July 1, 2018. 2018.

[8] Bureau of Economic Analysis. Gross Domestic Product by State, Fourth Quarter and Annual 2018. 2019.

[9] Medicaid and CHIP Payment and Access Commission. MACStats: Medicaid and CHIP Data Book December 2018. 2019.

[10] R Core Team. R: a language and environment for statistical computing. Vienna, Austria: R Foundation for Statistical Computing; 2022.

