# Supplementary material for: Differences in surgical site infection rates by state according to state-mandated operating room air changes per hour
Source: Antimicrob Resist Infect Control. 2025 Oct 8;14:115. doi: 10.1186/s13756-025-01631-5 (PMC12506489; doi:10.1186/s13756-025-01631-5)

Supplementary tables and figures

Table S1: Overall characteristics of states under ACH mandates, and those with missing information.

| Surgery type  Median [IQR]  N (%) | ACH mandate 15  (N=11) | ACH mandate 20  (N=36) | p-value^#^ | No ACH mandate data  (N=4*) |
| --- | --- | --- | --- | --- |
| State population 2018 (millions) | 2.9 [1.6, 5.3] | 5.0 [2.8, 8.6] | 0.09 | 5.8 [3.6, 8.8] |
| GDP 2018 (millions of US dollars) | 0.12 [0.07, 0.27] | 0.27 [0.12, 0.58] | 0.08 | 0.34 [0.22, 0.52] |
| Proportion on Medicaid | 20% [14%, 22%] | 23% [19%, 26%] | 0.1 | 23% [21%, 24%] |
| % SSI |  |  |  |  |
| Colon | 2.6 [2.4, 2.9]] | 2.4 [2.1, 2.5] | 0.06 | 2.0 [1.7, 2.0] |
| Hysterectomy | 0.6 [0.5, 0.7] | 0.6 [0.5, 0.7]] | 0.9 | 0.5 [0.5, 0.7] |
| Hip | 0.7 [0.6, 0.7] | 0.6 [0.5, 0.7]] | 0.6 | 0.5 [0.5, 0.5] |
| Knee | 0.4 [0.2, 0.4] | 0.4 [0.4, 0.5] | 0.2 | 0.4 [0.3, 0.4] |
| Rectal | - | 0.6 [0.3, 0.7] | - | 1.6 [1.6, 1.6] |
| Vag. Hysterectomy | 0.4 [0.3, 0.5] | 0.4 [0.2, 1.0] | 0.8 | 0.7 [0.7, 0.7] |
| CABG | 0.8 [0.6, 0.9] | 0.6 [0.6, 0.8] | 0.7 | 0.8 [0.7, 0.8] |
| Other cardiac | 0.3 [0.2, 0.5] | 0.3 [0.1, 0.4] | 0.9 | 0.4 [0.3, 0.5] |
| Bypass | 2.0 [2.0, 2.0] | 2.8 [1.9, 2.9] | 0.8 | 3.3 [2.8, 3.7] |
| Abdominal aortic aneurysm repair | - | 0.6 [0.3, 0.6] | - | - |
| C-section | 0.1 [0.1, 0.1] | 0.3 [0.2, 0.3]] | 0.04 | 0.2 [0.2, 0.3] |
| Spinal Fusion | 0.6 [0.4, 0.8] | 0.8 [0.7, 1.1] | 0.2 | 0.6 [0.5, 0.6] |
| Laminectomy | 0.7 [0.5, 0.8] | 0.4 [0.3, 0.5] | 0.1 | 0.3 [0.2, 0.4] |
| Gall bladder | - | 0.4 [0.3, 0.8] | - | - |
| Open Fracture - adult | 1.0 [1.0, 1.0] | 1.2 [0.9, 1.2] | 0.5 | 0.8 [0.7, 0.9] |

ACH: Air changes per hour, CABG: coronary artery bypass graft, GDP: gross domestic product, IQR: interquartile range, SSI: surgical site infection; *Hawaii, Ohio, Wisconsin, District of Columbia; ^#^for the comparison of ACH-15 and ACH-20 (excluding those missing).

Table S2: Estimates from fitted uni- and multivariable Poisson regression models comparing ACH-15 (reference) with ACH-20 mandates; an IRR<1 indicates reduced SSI risk for states with ACH-20 mandates.

| **Surgery type** | Univariable  (IRR [95% CI]) | p-value | Multivariable*  (aIRR [95% CI]) | p-value |
| --- | --- | --- | --- | --- |
| Colon | 0.88 [0.83, 0.94] | <0.001 | 0.88 [0.82, 0.94] | <0.001 |
| Hysterectomy | 0.90 [0.79, 1.02] | 0.1 | nE | nE |
| Hip | 0.98 [0.86, 1.11] | 0.7 | nE | nE |
| Knee | 1.08 [0.96, 1.23] | 0.2 | nE | nE |
| Rectal | - | - | - | - |
| Vag. Hysterectomy | 1.22 [0.39, 3.85] | 0.7 | nE | nE |
| CABG | 0.93 [0.76, 1.13] | 0.5 | nE | nE |
| Other cardiac | 0.64 [0.23, 1.73] | 0.4 | nE | nE |
| Bypass | 1.11 [0.55, 2.26] | 0.8 | nE | nE |
| Abdominal aortic aneurysm repair | - | - | - | - |
| C-section | 1.91 [1.14, 3.21] | 0.01 | nE^$^ | nE |
| Spinal Fusion | 1.74 [1.37, 2.22] | <0.001 | 1.92 [1.50, 2.46] | <0.001 |
| Laminectomy | 0.64 [0.43, 0.95] | 0.03 | - | 0.2 |
| Gall bladder | - | - | - | - |
| Open Fracture - adult | 0.84 [0.32, 2.26] | 0.7 | nE | nE |

aIRR: (adjusted) incidence rate ratio for the ACH mandate (reference ACH-15); CI: confidence interval; *adjusted for ACH mandate (15 [reference] or 20), state population, GDP, percentage number of persons on Medicaid; nE not estimated as ACH mandate not significant at the 5% level in univariable models (refer to methods); ^$^collinearity between GDP and ACH-20, multivariable model not estimable.

Figure S1: Percentage surgical site infection (SSI) for ACH mandate (15 and 20) for each surgery type.


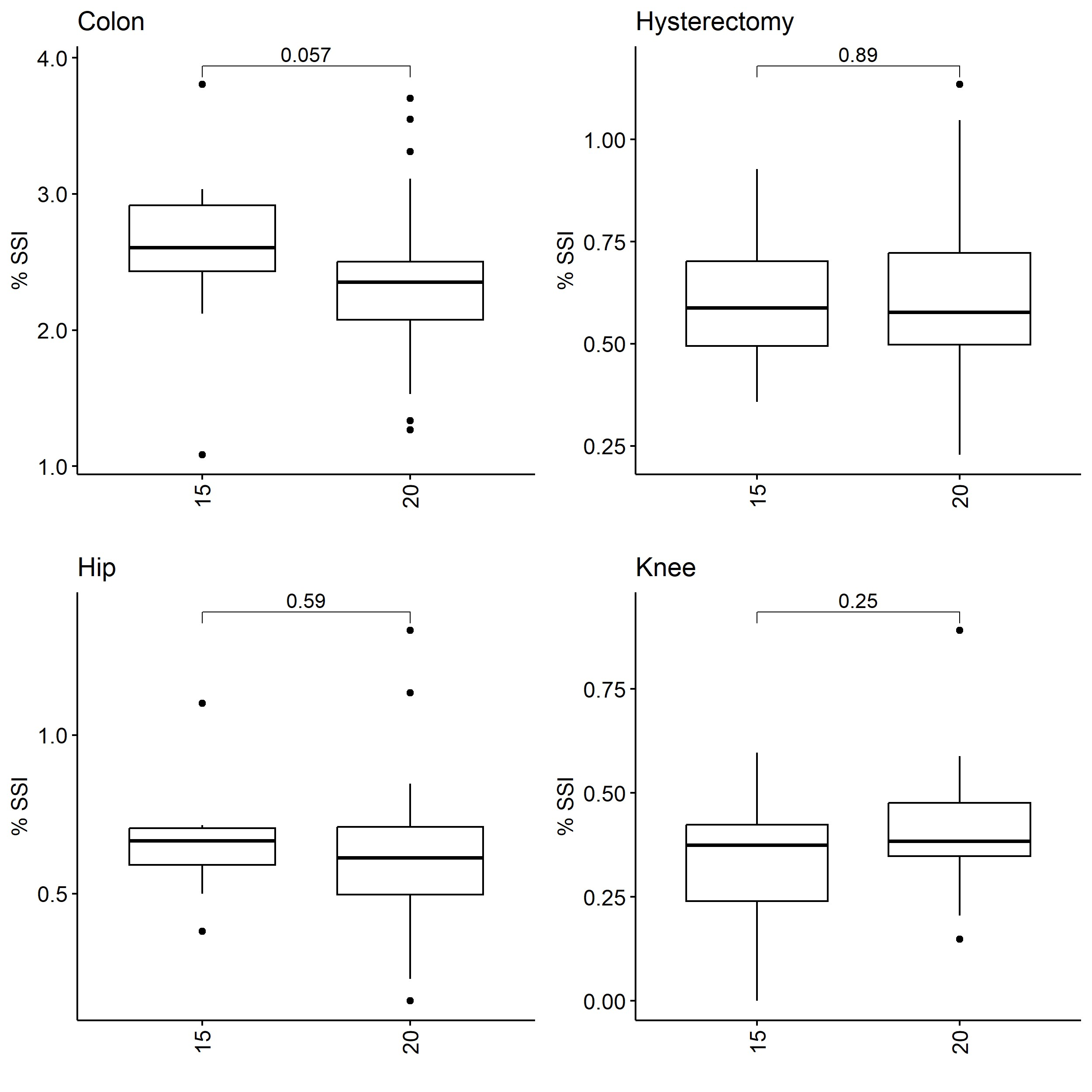


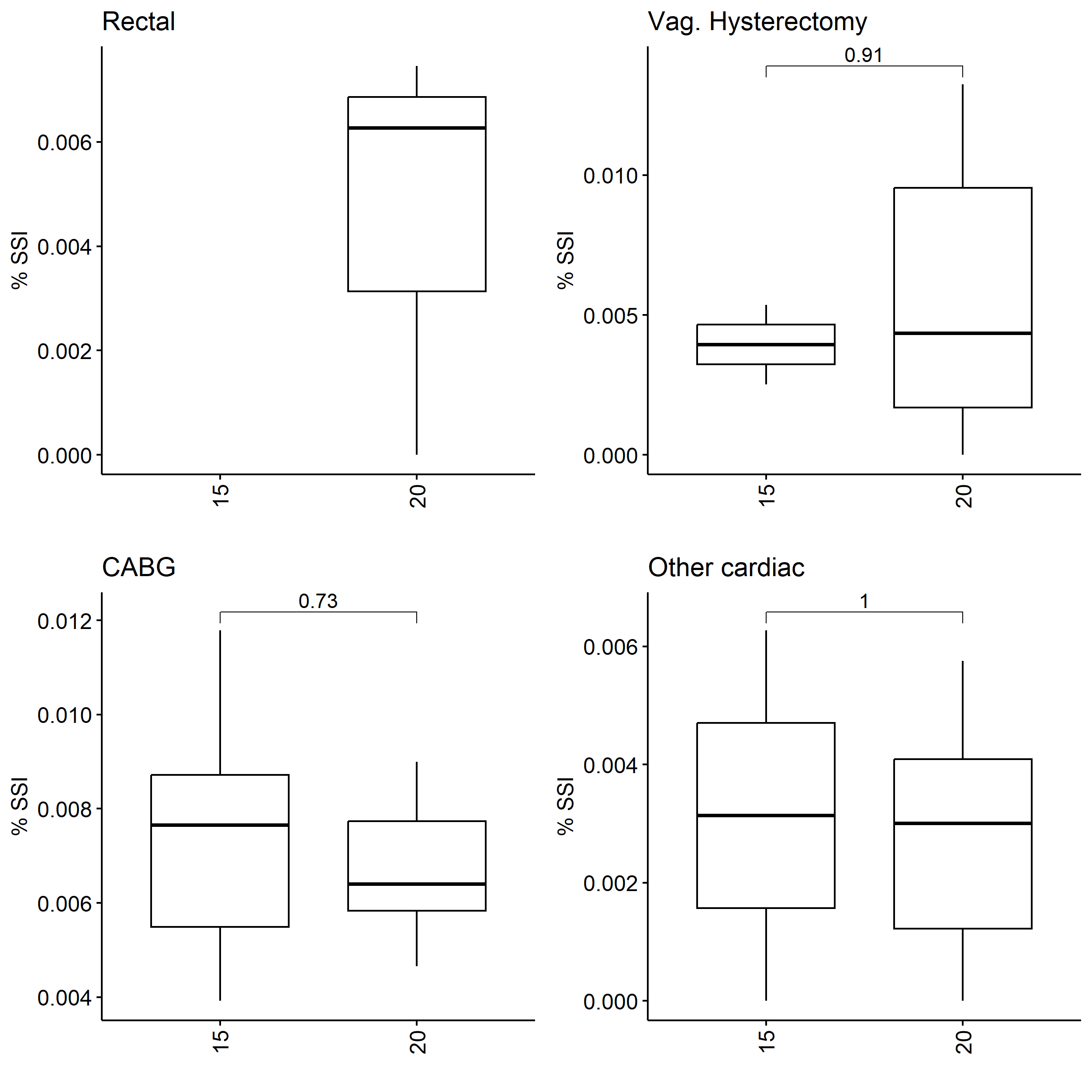


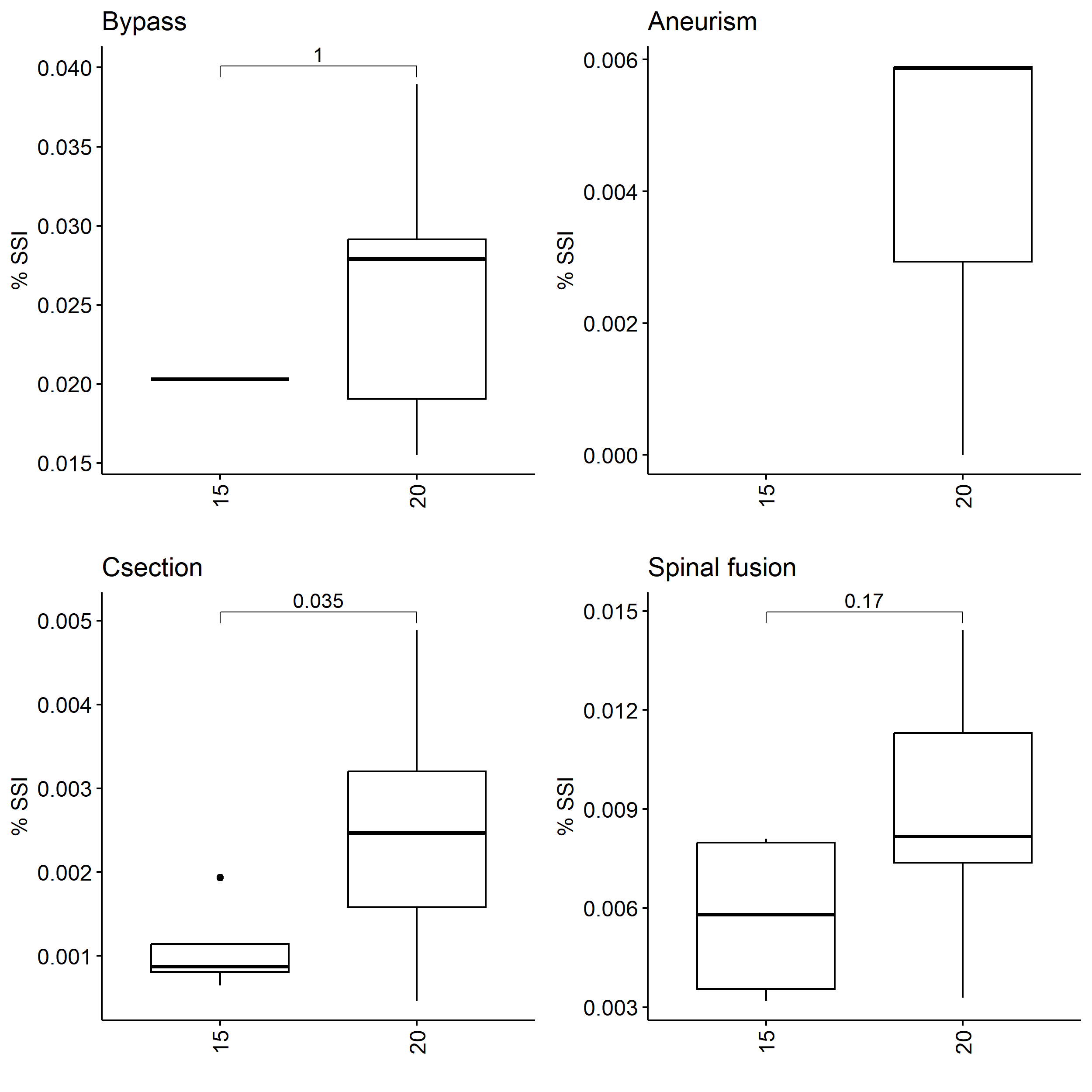


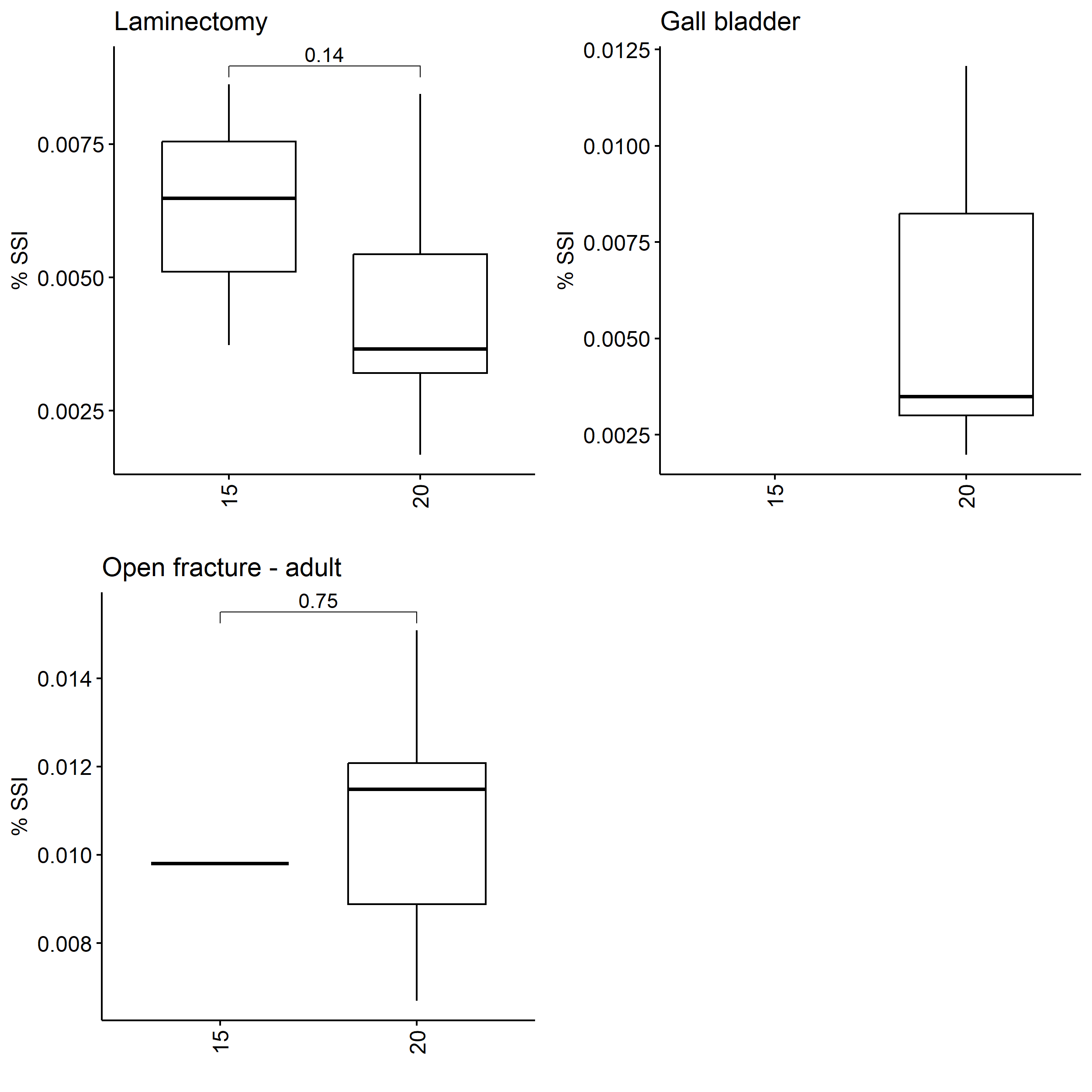

Supplement: Supplementary file 1 — Supplementary Material 1 [file 13756_2025_1631_MOESM1_ESM.docx]
